# Natural flavonoids derived from herbal medicines are potential anti-atherogenic agents by inhibiting oxidative stress in endothelial cells

**DOI:** 10.3389/fphar.2023.1141180

**Published:** 2023-02-24

**Authors:** Ruo-Lan Li, Ling-Yu Wang, Hu-Xinyue Duan, Die Qian, Qing Zhang, Li-Sha He, Xue-Ping Li

**Affiliations:** ^1^ Chengdu University of Traditional Chinese Medicine, Chengdu, China; ^2^ Hospital of Chengdu University of Traditional Chinese Medicine, Chengdu, China

**Keywords:** atherosclerosis, endothelial dysfunction, oxidative stress, herbal medicines, flavonoids

## Abstract

As the common pathological basis of various cardiovascular diseases, the morbidity and mortality of atherosclerosis (AS) have increased in recent years. Unfortunately, there are still many problems in the treatment of AS, and the prevention and treatment of the disease is not ideal. Up to now, the occurrence and development of AS can roughly include endothelial cell dysfunction, vascular smooth muscle cell proliferation, inflammation, foam cell production, and neoangiogenesis. Among them, endothelial dysfunction, as an early event of AS, plays a particularly important role in promoting the development of AS. In addition, oxidative stress occurs throughout the causes of endothelial dysfunction. Some previous studies have shown that flavonoids derived from herbal medicines are typical secondary metabolites. Due to its structural presence of multiple active hydroxyl groups, it is able to exert antioxidant activity in diseases. Therefore, in this review, we will search PubMed, Web of Science, Elesvier, Wliey, Springer for relevant literature, focusing on flavonoids extracted from herbal medicines, and summarizing how they can prevent endothelial dysfunction by inhibiting oxidative stress. Meanwhile, in our study, we found that flavonoid represented by quercetin and naringenin showed superior protective effects both *in vivo* and *in vitro*, suggesting the potential of flavonoid compounds in the treatment of AS.

## 1 Introduction

Cardiovascular disease (CVD) ranks alongside cancer, diabetes, and chronic respiratory diseases as the four diseases with the highest morbidity and mortality worldwide (Zhong et al., 2019). More than 17 million people die from CVD every year, accounting for more than 31% of global deaths (Townsend et al., 2016; Benjamin et al., 2017). Shockingly, with the acceleration of population aging, the incidence and mortality of CVD are still increasing, and there are large problems in the existing treatment methods need to be solved (Yusuf et al., 2004). Among them, atherosclerosis (AS), as the common pathological basis of CVD, has also received extensive attention in the prevention and treatment of CVD. AS is a chronic, progressive multifocal arterial disease, which mainly causes damage to large and medium-sized arteries. Unfortunately, although much effort has been invested in AS, the prevention and therapy of the disease are not particularly ideal (Falk, 2006; Crea and Libby, 2017). So far, the measures to alleviate AS have mainly been to reduce hyperlipidemia, slow the disease process, and mitigate the consequences of AS (Khan et al., 2021). Smoking, unhealthy diet, obesity, alcohol consumption and other factors may contribute to the development of AS. However, due to the complexity of AS, the AS pathogenesis is not well understood, which greatly reduced the therapeutic effect of AS (Steven et al., 2019). Based on the evidence from recent years, the occurrence and development of AS mainly involves a variety of cellular events such as endothelial cell dysfunction, vascular smooth muscle cell (VSMC) proliferation, inflammation, foam cell production, and neovascularization (Shemiakova et al., 2020). Pleasantly surprise, endothelial dysfunction appears to be reversible with therapeutic interventions aimed at correcting risk factors for endothelial dysfunction. At the same time, most of the initiation of AS development is located in the subendothelial space, and can be controlled by the endothelium and hormones. The treatment and improvement of endothelial dysfunction also play a particularly important role in AS. At present, there are many theories about the causes of endothelial cell dysfunction. Notably, inflammation, oxidative stress, autophagy and other events are inseparable from endothelial cell dysfunction, while oxidative stress is carried throughout (Li et al., 2022a).

For thousands of years, herbal medicines have been widely used in the prevention and treatment of diseases. With the development of medical information technology, flavonoids derived from herbal medicines have received more and more attention due to their significant efficacy and high safety (Li et al., 2022b). Flavonoids are mainly found in vacuoles of plants and are a secondary metabolite with abundant content. The main function of flavonoids is to protect plants against pathogens and UV radiation, and to participate in pollination by being recognized by pollinators (Pandey and Rizvi, 2009). Previous studies have shown that flavonoids have unique antioxidant activity due to their ability to provide hydrogen atoms or electrons, which can directly remove reactive oxygen species, thereby limiting the effects of oxidative stress (Li et al., 2022a). In addition, a large number of literature studies have shown that flavonoids derived from herbal medicines also have a significant effect on AS. Notably, flavonoids derived from herbal medicines also have been shown to regulate endothelial cell dysfunction during AS development (Yamagata, 2019). Therefore, based on the above explanation, we can propose that flavonoids derived from herbal medicines can inhibit oxidative stress, thereby inhibiting the occurrence of endothelial dysfunction.

## 2 Endothelial dysfunction contributes to the development of AS

**FIGURE 1:**
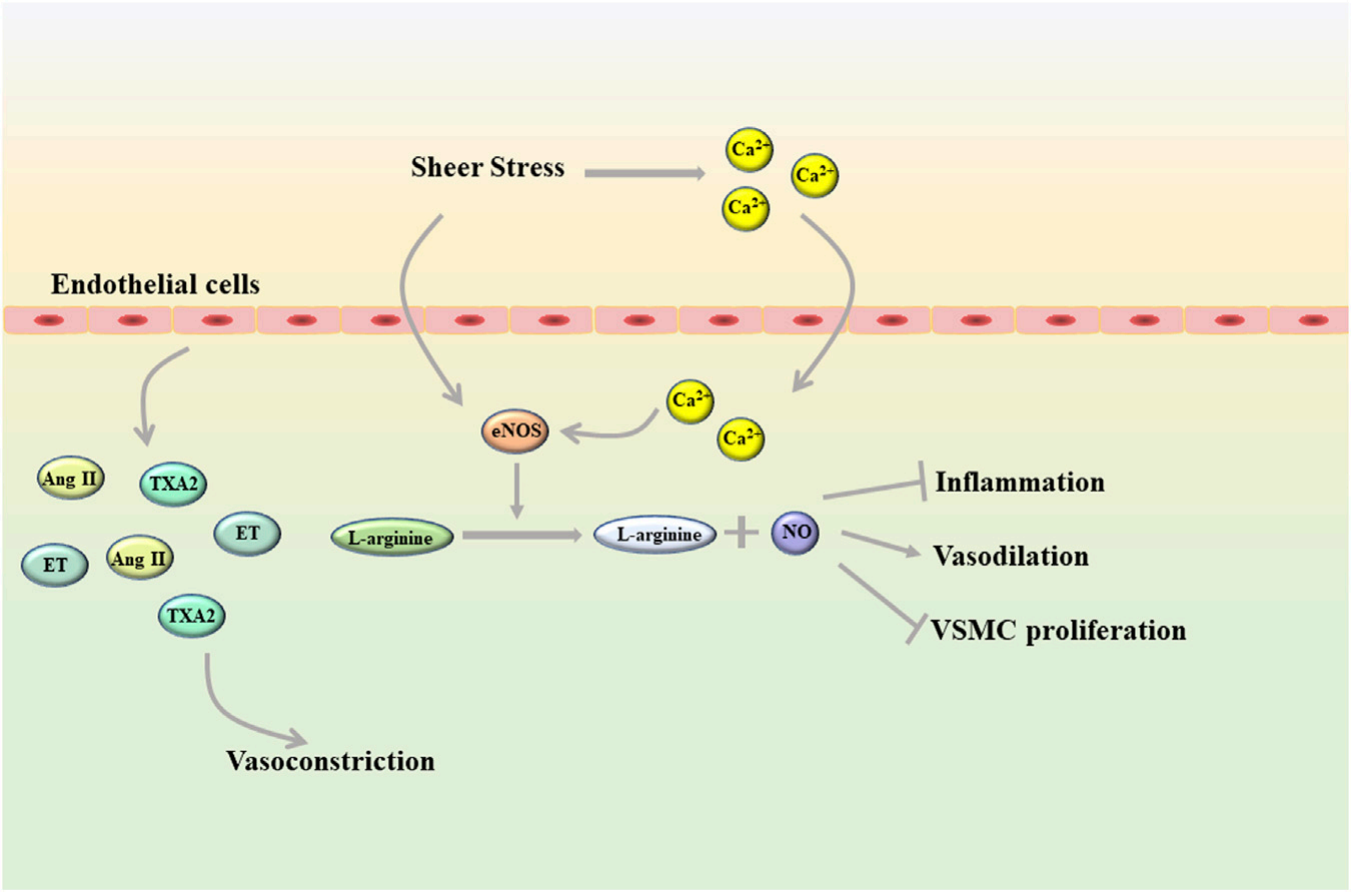
Shear stress helps endothelial cells maintain homeostasis in a healthy state (Ang II, angiotensin II; eNOS, endothelial NO synthase; ET, endothelin; TXA2, thromboxane A2; VSMC, vascular smooth muscle cells).

Endothelial cells, as a unique type of epithelial cells, are distributed in a monolayer of blood vessels and constitute the vascular endothelium that maintains vascular homeostasis (Krüger-Genge et al., 2019). The vascular endothelium is a semipermeable barrier between plasma and vascular tissue that extends along the entire circulatory system. Due to its unique location, endothelial cells can not only undergo metabolic exchange with plasma and interstitial fluid, but also interact with cells in the blood vessel wall (Yamaoka-Tojo, 2017). In addition, changes in blood composition and blood flow also have a great influence on the function of endothelial cells, among which mechanical transduction due to shear stress is considered to be the most important factor (Mitra et al., 2017; Mensah et al., 2020). In a healthy state, shear stress can directly promote the activation of endothelial NO synthase (eNOS) in endothelial cells, and also can activate eNOS by inducing rapid influx of Ca^2+^ into cells. eNOS promotes nitric oxide (NO) production by converting L-arginine to L-citrulline and NO (Förstermann and Munzel, 2006; Xu et al., 2021). As all we known, NO is an important vasoactive substance (Figure 1). NO can diffuse into vascular smooth muscle cells (VSMC), promote vasodilation by stimulating soluble guanyl cyclase and increasing cyclic guanosine monophosphate (cGMP), and has an antiproliferative effect on VSMC (Jin and Loscalzo, 2010). In the circulatory system, NO can also inhibit the adhesion and aggregation of platelets and exert anti-inflammatory properties. In addition, molecules represented by hydrogen sulfide (H_2_S), carbon monoxide, and arachidonic acid metabolites can also mediate vasodilation by inducing endothelium-derived hyperpolarization (Shimokawa, 2014). Under physiological conditions, in addition to vasodilation, endothelial cells can also mediate vasoconstriction by releasing a variety of vasoconstrictor molecules such as thromboxane A2 (TXA2), angiotensin (Ang) II and endothelin (ET) (Ley et al., 2007; Rao et al., 2007). Besides this, endothelial cells also can regulate platelet activity, coagulation cascade and fibrinolysis system. However, these functions of endothelial cells can be disrupted to varying degrees by diseases, including hyperlipidemia, diabetes, and heart failure (Tuñón et al., 2007; Tonelli et al., 2016; Giannitsi et al., 2019). Apparently, aging and genetic changes can also induce endothelial cell dysfunction.

Inflammation, oxidative stress and autophagy are considered as important cellular events that affect endothelial function. Previous studies have shown that lipids in endothelial cells can be transported to autophagic vesicles for lysosome-mediated degradation after ox-LDL stimulation. At the same time, ER stress is triggered in endothelial cells and further induces autophagy (Torisu et al., 2016). In addition, endothelial cells can also regulate autophagic flux through different transcription factors when shear stress is changed (Hua et al., 2022). Therefore, autophagy has also been proposed as an effective tool to alleviate endothelial dysfunction. Since inflammation is an important factor in inducing endothelial dysfunction, its role in AS cannot be ignored. When endothelial cells are activated, interleukin (IL) −8, chemokines, vascular adhesion molecule-1 (VCAM-1), growth factors and other inflammatory factors are secreted, attracting monocytes and neutrophils to adhere to endothelial cells and penetrate the arterial wall to cause inflammation (Chistiakov et al., 2018). There are many ways to induce endothelial inflammation. For example, lipopolysaccharide release from the blood promotes inflammation by increasing the expression of interferon-induced proteins and tetrapeptide repeats in endothelial cells (Wang et al., 2020). Insulin can increase Ang-II expression through the p38 MAPK-cFOS pathway and enhance inflammation in a paracrine manner (Chandel et al., 2020). In addition, excessive ROS can also induce endothelial dysfunction by enhancing inflammatory response (Zeng et al., 2020).

**FIGURE 2:**
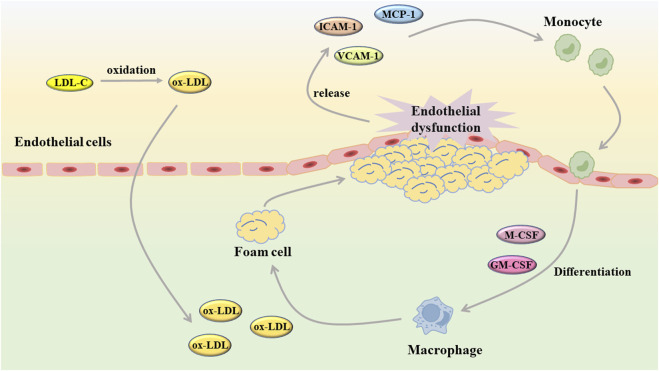
Endothelial dysfunction contributes to the development of atherosclerosis (GM-CSF: granulocyte macrophage colony stimulating factor; ICAM-1, intercellular cell adhesion molecule-1; LDL-C, low-density lipoprotein cholesterol; MCP-1, monocyte chemoattractant protein-1; M-CSF, macrophage colony stimulating factor; ox-LDL, oxidized low-density lipoprotein; VCAM-1, vascular cell adhesion molecule-1).

When endothelial injury occurs in blood vessels, white blood cells will combine with fibrin tissue to form fibrin network, which plays a role in the repair of endothelial injury (Ley et al., 2007; Rao et al., 2007). Unfortunately, when the body suffers from a wide range of pathological damage, vascular endothelial cells are continuously damaged and stimulated, and the repair effect is ineffective. Under these conditions, the endothelial cells undergo a phenotypic shift, the balance between vasodilator and vasoconstrictor is disrupted, and the arterial structure is destroyed (Incalza et al., 2018; Kim et al., 2019). As an early event of AS, endothelial cell dysfunction plays a role in the development of AS (Figure 2). After the occurrence of endothelial cell dysfunction, the vascular barrier function is weakened, and the low-density lipoprotein cholesterol (LDL-C) in the blood is more likely to accumulate in the intima and undergo oxidation reaction, and then produce oxidized low-density lipoprotein (ox-LDL) (Gao et al., 2017). The injured endothelial cells will release monocyte chemoattractant protein-1 (MCP-1), intercellular cell adhesion molecule-1 (ICAM-1), vascular cell adhesion molecule-1 (VCAM-1) and so on to induce monocyte and macrophages to adhere to the vessel wall (Clapp et al., 2004; Chi and Melendez, 2007). Subsequently, macrophage colony stimulating factor (M-CSF) and granulocyte macrophage colony stimulating factor (GM-CSF) stimulate mononuclear macrophages to differentiate into macrophages, which will take up ox-LDL to generate foam cells and further aggravate AS (Trus et al., 2020; Zhi et al., 2023). As an important component of vascular composition, VSMC will switch from a contractile to a synthetic phenotype after endothelial cell injury. Similarly, VSMC also undergo abnormal proliferation and migration induced by chemokines and matrix metalloproteinases (MMP), which destroys the stability of plaques. In the intima, VSMC not only uptake ox-LDL to generate foam cells, but also secrete extracellular matrix components to form fibrous caps (Liang et al., 2018; Muqri et al., 2020).

### 3 Oxidative stress and endothelial dysfunction in AS

As mentioned above, factors such as hyperlipidemia, diabetes, heart failure, aging, and genetics may contribute to the development of endothelial dysfunction. Among these factors, we can find the presence of oxidative stress and ox-LDL. At present, many studies believe that excessive reactive oxygen species (ROS) can induce oxidative stress on the one hand, and aggravate the oxidative modification of LDL on the other hand. Subsequently, oxidative stress interacts with ox-LDL to jointly promote the occurrence of endothelial dysfunction.

### 3.1 Mechanisms of ROS generation

**FIGURE 3:**
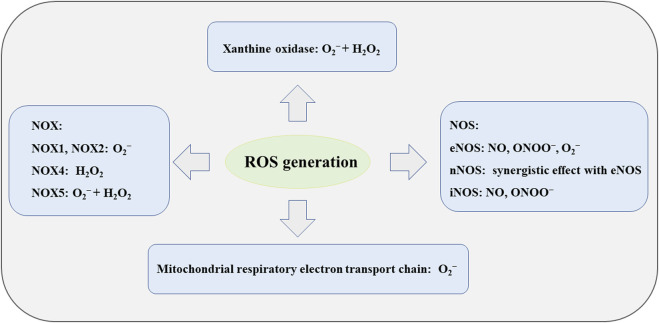
The generation of ROS.

ROS is an endogenous and important mediator involved in various biological processes of the organism and can serve as a second messenger in cell signaling. Because ROS can easily acquire or loss electrons, it is widely involved in redox reactions. However, when the content of ROS exceeds limitation, it will disrupt the redox balance in the body, which in turn leads to the occurrence of oxidative stress, thereby affecting all aspects of physiological functions (Kattoor et al., 2017). Nowadays, the ROS family includes many small molecules and ions, such as superoxide, hydroxyl radicals, hydrogen peroxide and so on. It is well known that almost all cells in blood vessels can produce ROS, and its generation mechanism mainly includes NADPH oxidase (NOX), xanthine oxidase, mitochondrial respiratory chain and NOS (Figure 3) (Goszcz et al., 2015).

As a membrane-binding enzyme complex, NOX is the only family of enzymes whose main function is to produce ROS. NOX is widely present in various vascular cells and is the main source of ROS by transferring electrons from NADPH to O_2_ and generate O_2_^−^. When the body has hypertension, diabetes or high cholesterol, it is easy to increase the expression of NOX and thus increase the content of ROS in the body (Balaban et al., 2005). Existing studies have shown that the congeners of NOX are expressed in various types of vascular cells, but the difference in their content cannot be ignored. For example, NOX2, NOX4, and NOX5 are predominantly expressed in EC, whereas NOX1 and NOX4 are predominantly expressed in VSMC. Different NOXs produce different types of ROS, with NOX1 and NOX2 generating O_2_^−^, NOX4 generating H_2_O_2_, and NOX5 generating O_2_^−^ and H_2_O_2_ (Dikalov et al., 2008; Lassègue et al., 2012). At the same time, different NOX have different effects on AS. For example, downregulation of NOX1, NOX2, and NOX5 can inhibit AS, while NOX-4 has a cardioprotective effect (possibly due to the fact that NOX4 mainly produces H_2_O_2_) (Guzik et al., 2008; Takac et al., 2011; Fulton and Barman, 2016). Xanthine oxidase is another important enzymatic source of ROS and is mainly present in EC. Xanthine oxidase can generate O_2_^−^ and H_2_O_2_ by oxidation of xanthine and hypoxanthine. In addition, xanthine oxidase can also elevate LOX-1 and CD-36 in macrophages and VSMCS, disrupt intracellular lipid metabolism, and increase the risk of AS (Kattoor et al., 2017).

Mitochondria, as an important organelle within the cells, is an important source of energy required for cellular activities. Oxygen, which is required for cell survival, is converted to O_2_^−^ in the mitochondrial respiratory electron transport chain mainly by electron grant in complexes I, II, and III for energy production and oxidative phosphorylation (Peng et al., 2019; Peoples et al., 2019). This process is recognized as the main way to generate ROS. Normally, ROS generated by this process can be removed by various oxidoreductases to maintain homeostasis. Under pathological conditions, the disruption of this balance will lead to excessive accumulation of ROS and further induce ROS leakage (Peoples et al., 2019).

NOS has three distinct isoforms, namely, neuronal NOS (nNOS), inducible NOS (iNOS) and endothelial NOS (eNOS). Among them, eNOS is most closely associated with AS. Notably, although eNOS could produce NO in the presence of tetrahydrobiopterin (BH4) to scour oxygen radicals and thus exert anti-atherosclerosis effect, it has been shown in previous studies that overexpression of eNOS may also promote the development of AS. The possible mechanism lies in the decoupling of eNOS caused by excessive BH4 depletion (Ozaki et al., 2002; Hossain et al., 2012). This hypothesis has been confirmed by a recent study. It was shown that when BH4 was scarce, eNOS uncouples to generate O_2_^−^ and combines with NO to generate peroxynitrite (ONOO^−^). ONOO^−^ is a potent oxidant that induces the occurrence of oxidative stress damage (Li et al., 2015). nNOS can exert a synergistic effect with eNOS in anti-atherosclerosis by regulating vascular tone (Capettini et al., 2011). However, iNOS can not only induce excessive production of NO, but also compete with eNOS to bind BH4, promote the generation of ONOO^−^, and aggravate the occurrence and development of AS (Gunnett et al., 2005).

### 3.2 ROS promotes ox-LDL production and aggravates endothelial dysfunction

It was shown that excessive ROS-induced oxidative stress can directly affect intracellular biomacromolecules to cause damage. ROS and its oxidation products can act as signal transduction molecules to activate related pathways, damage endothelial cells, and promote the development of AS.

As one of the oxidation products, ox-LDL is thought to play a major role in lipid metabolic disorders. LDL-related modifications include oxidation, deacetylation, glycosylation and aggregation, among which the oxidation of LDL is closely related to AS (Nègre-Salvayre et al., 2017). ROS can oxidise a variety of polyunsaturated lipids in blood vessels, and the by-products formed can react with apolipoprotein B-100 and damage its function. Subsequently, modified ApoB-100 retards LDL removal and prolongs the exposure of lipids and apoB-100 to ROS attack, which further enhances LDL oxidation (Negre-Salvayre et al., 2008; Rabbani et al., 2010; Nègre-Salvayre et al., 2017). When endothelial cells are exposed to oxidative stress for a long time, their structure and function are continuously damaged, which also leads to the continuous oxidation of LDL to form ox-LDL (Stocker and Keaney, 2004). However, after numerous studies on the oxidation mechanism of LDL, it has been found that ox-LDL is heterogeneous, and different concentrations of ox-LDL also have a dual effect on vascular cells. For example, low concentrations of ox-LDL can induce cell migration and proliferation, and create a pro-inflammatory environment for AS, while high concentrations of ox-LDL can promote apoptosis (Dandapat et al., 2007; Cinq-Frais et al., 2013; Camaré et al., 2015). Excessive ROS can cause endothelial cell apoptosis through several major pathways. Firstly, ROS can not only activate nuclear factor kappa-B (NF-κB) through redox factor-1 (Ref-1), but also directly activate NF-κB. Subsequently, activated NF-κB translocates into the nucleus where it binds to the apoptosis-related gene c-Myc, promoting gene transcription and inducing apoptosis. The p38 pathway and c-Jun N-terminal kinase pathways are also strongly associated with ROS-induced apoptosis (Haghi Aminjan et al., 2019; Zhang L. et al., 2022). Notably, excessive ROS causes lipid peroxidation, damages the inner mitochondrial membrane, and ultimately induces both endogenous and exogenous endothelial cell apoptosis (Sinha et al., 2013). In addition, the generated ox-LDL disrupts the structure of actin filaments upon contact with endothelial cells, causing disruption of the cytoskeleton, which in turn alters endothelial cell permeability. The increased permeability of endothelial cells makes it easier for lipids to pass through the cells, further aggravating the development of AS (Chouinard et al., 2008; Zhang et al., 2022).

Ox-LDL can enter endothelial cells through a variety of cell-surface expressed scavenger receptors, the most typical of which are LOX-1 and CD36 (Nègre-Salvayre et al., 2017). LOX-1 is the main receptor for ox-LDL uptake by endothelial cells. The combination of LOX-1 and ox-LDL can enhance the expression of NOX, promote the generation of O_2_^−^, and aggravate the oxidative stress response in cells (Lu et al., 2011; Yoshimoto et al., 2011). At the same time, the oxLDL/LOX-1/ROS axis is activated, which promotes the production of various inflammatory cytokines, chemokines, adhesion molecules, and ultimately leads to the recruitment and adhesion of monocytes to the activated endothelium (Kamei and Carman, 2010; Lubrano and Balzan, 2016). As a multifunctional receptor, CD36 recognizes oxidized phospholipids and other ligands in addition to ox-LDL. When ox-LDL binds to CD36, MAPK, NF-κB and Toll-like receptors (TLR) are activated, which enhance the local response (Park et al., 2013).

## 4 Natural flavonoids derived from herbal medicines are potential anti-AS agents by inhibiting oxidative stress in endothelial cells

Flavonoids are a class of secondary metabolites widely found in plants and fungi. Their characteristic structure mainly contains 15 carbon atoms. Flavonoids can be subdivided according to their structure into anthocyanins, flavonoids, flavanones, flavonols, anthoxanthins, and isoflavonoids. Because flavonoids have hydroxyl groups in their structure, they can play an antioxidant role both *in vivo* and *in vitro*. In this review, we searched the relevant literature on flavonoids inhibiting oxidative stress to treat endothelial dysfunction in AS, and selected some important compounds to elaborate.

Quercetin is a natural polyhydroxy flavonoid found in a variety of plants such as *Bupleurum chinense* DC, *Bupleurum scorzonerifolium* Willd (Apiaceae), mulberry leaves, *Crataegus pinnatifida* Bunge, and *C. pinnatifida* var. *Major* N. E. Br. It is a plant secondary metabolite with antioxidant activity (Zhi et al., 2023). In the past decades, quercetin has been widely used in clinical practice for various diseases due to its superior activity, including cancer, arthritis, neurodegenerative diseases and cardiovascular diseases (Wang et al., 2022). There are numerous studies on quercetin in the treatment of AS. *In vivo* and *in vitro* studies have shown that quercetin exerts multiple effects on various processes of AS development, including foam cell formation, lipid metabolism, monocyte migration, and endothelial cell dysfunction. Firstly, intragastric administration of quercetin ameliorated arterial lipid deposition in high-fat diet fed ApoE mice. In ox-LDL-induced human umbilical vein endothelial cells (HUVECs), quercetin reduced intracellular ROS and increased mitochondrial membrane potential. At the same time, apoptosis and senescence induced by ox-LDL were also alleviated, lipid droplet deposition was reduced, and cell morphology was improved. By exploring the underlying mechanism, p53 and mTOR signaling pathways were found to be involved in the pharmacological mechanism of quercetin (Jiang et al., 2020).

Naringenin, a flavonoid extracted from the pericarp of *Citrus reticulata* Blanco, is a trihydroxy flavanone. It can be found in past reports that naringenin exerts antioxidant activity directly through free radical scavenging activity, and has the ability to induce endogenous antioxidant system (Hernández-Aquino and Muriel, 2018). The comparison of the antioxidant capacity of naringenin with that of quercetin has been controversial in some studies. It was reported that naringenin equivalent antioxidant activity was 1.53 mmol/L, a small value compared to the 4.7 mmol/L of quercetin (Rice-Evans et al., 1996). However, in the study of Cavia-Saiz et al., the antioxidant capacity of naringenin was worse than that of quercetin (Cavia-Saiz et al., 2010). Therefore, further studies are needed to compare the antioxidant capacity of naringenin with other flavonoids. However, it was no doubt about the role of naringenin in protecting endothelial dysfunction in AS. In previous experiments, naringenin was found to inhibit AS by ameliorating dyslipidemia, and subsequently it was found to protect mitochondrial membrane potential to ameliorate ischemic damage (Mulvihill et al., 2010; Testai et al., 2017). Therefore, in the study of Li et al., it was hypothesized that naringenin could ameliorate endothelial injury through a mitochondria-dependent pathway. After homocysteine-induced HUVECs injury, naringenin could inhibit the generation of ROS in mitochondria and cytoplasm, restore mitochondrial membrane potential, but there was no significant difference in Ca^2+^ concentration. RNA-seq transcriptome analysis and experimental validation showed that naringenin significantly restored the expression of Sirt1, AMPKα and eNOS. In addition, knockdown of Sirt1 and AMPKα by siRNA almost abolished this protective effect (Li et al., 2021). *In vivo*, endothelial injury was defined as plasma homocysteine levels higher than 15 μmol/L. Naringenin could significantly inhibit the damage of arterial wall and protect endothelial function after treatment, and its mechanism was consistent with the results *in vitro* (Li et al., 2021). Therefore, we can conclude that naringenin ameliorates homocysteine-induced endothelial injury through the AMPKα/Sirt1 pathway.

*Carthamus tinctorius* L. has been used as a traditional medicinal plant for thousands years. According to Kaibao Materia Medica, the dried flowers of *C. tinctorius* L. can promote blood circulation and relieve pain. So far, *C. tinctorius* L. has been developed as *Danhong* injection, safflower injection and other preparations for the treatment of coronary heart disease and angina pectoris. Hydroxysafflor yellow A is an important active component of *C. tinctorius* L., and it is also the most abundant component of safflower yellow, an indicator component of *C. tinctorius* L (Xue et al., 2021). In recent years, hydroxysafflor yellow A has been shown to protect endothelial cells by inhibiting inflammation and apoptosis. First, Ji et al. found that hydroxysafflor yellow A could increase the ratio of Bcl-2/Bax at the mRNA and protein levels and reduce mitochondrial-dependent apoptosis in hypoxia-induced HUVECs (Ji et al., 2009). This phenomenon was further illustrated in the experiments of Xie et al., which showed that hydroxysafflor yellow A could regulate cell survival and proliferation by promoting AKT and inhibiting PTEN expression. Meanwhile, hydroxysafflor yellow A reduced ROS generation and restored intracellular redox balance by increasing intracellular superoxide dismutase (SOD) in H_2_O_2_-induced HUVECs (Xie et al., 2020). In addition, in ox-LDL-induced HUVECs, hydroxysafflor yellow A could upregulate VDAC2 or inhibit apoptosis through AMPK signaling, in which VDAC2 could exert an anti-apoptotic effect by interfering with Bak-mediated apoptosis (Ye et al., 2017; Zhang H. et al., 2022).

Genistein is a natural isoflavone first obtained from *Genista tinctoria* L. It is mainly derived from *Euchresta japonica* Hook. f. ex Regel, *Sophora japonica* L. and so on. Currently, methanol, ethanol, acetonitrile and other organic solvents are used to extract genistein. Meanwhile, the chemical synthesis of genistein is simple and feasible (Spagnuolo et al., 2015). The structure of genistein is similar to that of endogenous estrogen, so it can bind to estrogen receptors and exert estrogen-like effects after being absorbed by the body. In addition, as a typical flavonoid, it is connected with multiple hydroxyl groups on the phenyl ring, which makes it have excellent antioxidant effects and can be applied to the treatment of cardiovascular diseases, diabetes, depression and other diseases (Borrás et al., 2006; Jafari et al., 2022). In endothelial dysfunction, genistein can effectively inhibit ROS and malondialdehyde (MDA) in cells, and restore the four oxidoreductases activities including superoxide dismutase (SOD), catalase (CAT), glutathione (GSH) and glutathione peroxidase (GPx). In this way, the redox balance of endothelial cells is maintained (Zhang et al., 2017). Further exploration revealed that the antioxidant activity of genistein was closely related to MR-34a/sirtuin-1/foxo3a. Genistein can downregulate the expression of MiR-34a in ox-LDL-induced HUVES, thereby promoting the expression of sirtuin-1. In addition, sirtuin-1 is known to exert antioxidant activity by activating fxo3a in previous studies. However, after genistein treatment, the expression of fxo3a was significantly increased (Zhang et al., 2017).

Baicalein, also known as 5, 6, 7-trihydroxyflavone, is a well-recognized natural flavonoid with antioxidant and anti-inflammatory activities. Baicalein is the most abundant component in the root of Scutellaria baicalensis (*S. baicalensis*) Georgi, a traditional Chinese medicine (also known as *Huangqin* in Chinese) (Huang et al., 2005). In a previous study, it was shown that baicalein inhibited IL-1β-induced ICAM-1 expression in HUVECs, suggesting that baicalein could protect endothelial cell function (Kimura et al., 1997). In a recent study, ox-LDL was used to induce apoptosis in HUVECs and baicalein was preincubated before induction. It was showed that baicalein effectively inhibited the generation of intracellular ROS and the release of cytochrome C from mitochondria, and increased mitochondrial membrane potential. The expression of pro-apoptotic protein BAX was downregulated, while the expression of anti-apoptotic protein Bcl-2 was upregulated. In addition, the bioavailability of NO was also improved (Chan et al., 2016). Subsequently, it was also shown that baicalein pretreatment could inhibit the binding ability of ox-LDL by reducing the expression of LOX-1, thereby inhibiting the generation of ROS. In addition, baicalein inhibited the protein expression of NADPH oxidase and increased the phosphorylation level of AMPK, thereby inhibiting the activation of protein kinase C (PKC)-α and PKC-β (Tsai et al., 2016).

Luteolin is a common flavonoid, which is usually found in the form of glycosylated in celery, green pepper, *Perilla frutescens* (L.) Britt., and *Matricaria recutita* L. Luteolin possesses the antioxidant properties, as well as anti-inflammatory ability. Therefore, it also has a good advantage in the treatment of AS (Prasher et al., 2022). Up to now, the antioxidant activity of luteolin has been fully confirmed. It can exert efficacy in all stages of AS, such as VSMC migration and proliferation, cell adhesion molecule secretion and endothelial cell dysfunction (Luo et al., 2017). When endothelial cells are dysfunctional, luteolin can inhibit the generation of intracellular ROS, while the phosphorylation of p38MAPK and nuclear translocation of NF-kB induced by ox-LDL are reversed. At the same time, the mRNA levels of ICAM-1, VCAM-1, selectin, MMP-1, MMP-2, and MMP-9 are also downregulated by luteolin (Yi et al., 2012). In another study, this conclusion was further developed. In other words, luteolin inhibited TNF-α-induced transcriptional activities of NF-κB and p38 as well as ERK1/2 phosphorylation, while it also exerted its inhibitory effect on Nox4 expression. Ultimately, luteolin restored the redox balance in endothelial cells, that is, the contents of GSH and SOD were restored and LDH was decreased (Xia et al., 2014).

*Erigeron breviscapus* (Vant.) Hand.-Mazz is a traditional natural medicine used to treat heart and brain ischemic diseases. The modern pharmacological studies have shown that the main active substance is scutellarin. Scutellarin, also known as 4′, 5, 6-trihydroxyflavone-7-glucuronde, is a member of the natural flavonoid family. Previous studies have found that scutellarin not only prevents cerebral ischemia by inhibiting inflammatory response, but also improves liver damage by inhibiting oxidative stress (Yuan et al., 2016). In addition, scutellarin also plays a role in endothelial dysfunction through its antioxidant effect in AS. Scutellarin scavenged excess ROS and increased the bioavailability of NO in HAECs induced by either angiotensin II or H_2_O_2_. The contents of oxidoreductases, including SOD, GPx, CAT and Nox, could be restored to varying degrees after treatment with scutellarin. Subsequently, the mechanism of scutellarin against endothelial cell injury and apoptosis was further studied, and the results showed that the protective effect of scutellarin was closely related to Hippo-FOXO3A and PI3K/AKT signaling pathways. After treating with scutellarin, the mRNA levels of mammalian sterile-20-like kinases 1 (Mst1), Yes-associated protein (YAP) and FOXO3A were significantly downregulated, as well as the protein levels of p-Mst1, p-YAP and nuclear translocation of FOXO3A. At the same time, PI3K/AKT signaling pathway was activated, and its downstream apoptosis-related Bax and Bcl-2 proteins were also changed (Mo et al., 2018; Fu et al., 2019). We can draw the same conclusion *in vivo* that scutellarin can alleviate lipid metabolism disorder and maintain redox balance in AS rats through Hippo-FoxO3A and PI3K/AKT signaling pathways (Fu et al., 2019). The specific indicators are referred to Table 1.

**TABLE 1:** Natural flavonoids derived from herbal medicines are potential anti-AS agents by inhibiting oxidative stress in endothelial cells.

Components	Plant source	Structure	Experimental model	Effective dose	Effect and mechanism	Ref
Quercetin	*Bupleurum chinense* DC, *Bupleurum scorzonerifolium* Willd (Apiaceae), mulberry leaves, *Crataegus pinnatifida Bunge, Crataegus pinnatifida* var. *Major* N. E. Br	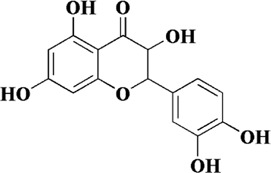	Ox-LDL-induced HUVECs	0.3, 1, 3 μM	MMP↑	Jiang et al. (2020)
					ROS↓, lipid droplet deposition↓, p53↓, mTOR↓	
			High-fat diet fed ApoE mice	20 mg/kg/d	Arterial lipid deposition↓	
Naringenin	*Citrus reticulata* Blanco	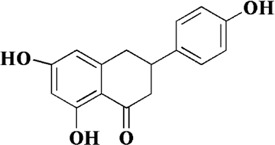	Homocysteine-induced HUVECs	200 μM	MMP↑, the mRNA of AMPKα and Sirt1↑, the protein of AMPKα, Sirt1 and eNOS↑	Li et al. (2021)
					ROS↓, cytoplasmic cytochrome c↓	
			High-methionine induced SD rat	100 mg/kg/d	SOD↑, NO↑, AMPKα↑, Sirt1↑, eNOS↑	
					Homocysteine↓, MDA↓	
Hydroxysafflor yellow A	*Carthamus tinctorius* L	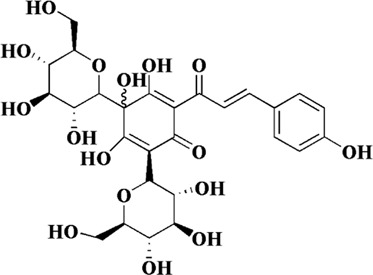	Hypoxia-induced HUVECs	1, 10, 100 μM	Bcl-2/Bax↑, eNOS↑ p53↓	Ji et al. (2009)
			Ox-LDL-induced HUVECs	50 μM	SOD↑, NO↑, NOX4↑, AMPKα↑, p-AMPKα↑	Zhang et al. (2022b)
					ROS↓	
			Ox-LDL-induced HUVECs	1, 5, 25 μM	NO↑, VDAC2↑	Ye et al. (2017)
					SOD↓	
			H_2_O_2_-induced HUVECs	4 and 8 μg/mL	GSH/GSSG↑, SOD↑, Bcl-2↑, AKT↑	Xie et al. (2020)
					ROS↓, Bax↓, PTEN↓	
Genistein	*Euchresta japonica* Hook. f. ex Regel, *Sophora japonica* L. and so on	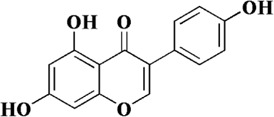	Ox-LDL-induced HUVECs	10, 100, 1,000 nM	SOD↑, CAT↑, GSH↑, GPx↑, sirtuin-1↑, foxo3a↑	Zhang et al. (2017)
					MiR-34a↓	
Baicalein	Scutellaria baicalensis (*S. baicalensis*) Georgi	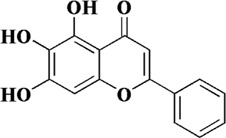	Ox-LDL-induced HUVECs	2.5–20 μM	NO↑, cytochrome C in mitochondria↑, mitochondrial membrane potential↑, Bcl-2↑	Chan et al. (2016)
					ROS↓, BAX↓	
			Ox-LDL-induced HUVECs	2.5 μM	p-MAPK↑	Tsai et al. (2016)
					LOX-1↓, NADPH↓, p-PKC-α↓, p-PKC-β↓, p47phox↓, Rac-1↓	
Luteolin	Celery, green pepper, *Perilla frutescens* (L.) Britt., and *Matricaria recutita* L	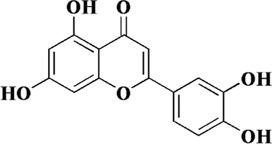	Ox-LDL-induced EA.hy926	40 μM	p-p38MAPK↑, nuclear translocation of NF-kB↑	Yi et al. (2012)
					ROS↓, ICAM-1↓, VCAM-1↓, selectin↓, MMP-1↓, MMP-2↓, MMP-9↓	
			TNF-α-induced HUVECs	6.25, 12.5, 25 μM	GSH↑, SOD↑, Bcl-2↑	Xia et al. (2014)
					ROS↓, LDH↓, NF-κB↓, p38↓, p-ERK1/2↓, ICAM-1↓, VCAM-1↓, Nox4↓	
Scutellarin	*Erigeron breviscapus* (Vant.) Hand.-Mazz	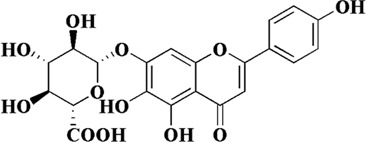	H_2_O_2_-induced HUVECs	12.5, 50, 200 μM	SOD1↑, NO↑, SOD↑, GPx↑, CAT↑	Mo et al. (2018)
					MDA↓, Ca^2+^↓, Nox4↓	
			Angiotensin II -induced HUVECs	50, 100, 200 μM	SOD↑, CAT↑, Bcl-2↑, PI3K↑, p-AKT↑	Fu et al. (2019)
					MDA↓, ROS↓, Caspase-3↓, FAS↓, BAX↓, Bim↓, p-Mst1↓, p-YAP↓, p-FOXO3A↓	
			HFD-induced rat	6.25 and 25 mg/kg/d	HDL↑, IL-1α↑, SOD↑, CAT↑, Bcl-2↑, PI3K↑, p-AKT↑	
					TG↓, TC↓, LDL↓, VCAM-1↓, ICAM-1↓, IL-6↓, TNF-α↓, MDA↓, Caspase-3↓, Fas↓, Bim↓, Bax↓, p-Mst1↓, p-YAP↓, p-FOXO3A↓, FOXO3A↓	
Acacetin	*Robinia pseudoacacia L., Dendranthema morifolium* (Ramat.)Tzvel., and *Saussurea involucrata* (Kar. et Kir.) Sch.-Bip	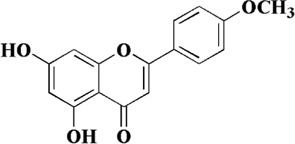	High glucose-induced HUVECs	0.3, 1, 3 μM	MMP↑, SOD↑, mitoBcl-2/mitoBax↑, Sirt3↑, pAMPK↑, PGC-1α↑	Han et al. (2020)
					ROS↓, MDA↓	
			Streptozotocin-induced diabetic ApoE^−/−^ mice	20 mg/kg/d	SOD1↑, SOD2↑, Sirt1↑, PGC-1α↑, Sirt3↑, pAMPK↑, Bcl2↑	
					TG↓, TC↓, LDL↓, lipoprotein A↓, lipoprotein B↓, Bax↓	
			Ox-LDL-induced EA.hy926	3 μM	Bcl-2↑, MsrA↑, Nrf2↑, HO-1↑, CAT↑	Wu et al. (2021)
					ROS↓, Bax↓, caspase-3↓, Keap1↓	
Eupatilin	*Artemisia princeps* Pampanini	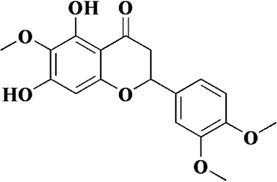	TNF-α-induced HUVECs	6.25, 12.5, 25 μM	ROS↓, VCAM-1↓, ICAM-1↓, NF-kB p65↓, p-MAPK↓	Yu et al. (2015)
Apigenin	*Clinopodium chinense* (Benth.) O. Kuntze	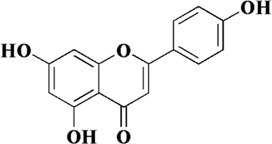	High glucose-induced HUVECs	3, 30 μM	NO↑, p-Akt↑, Bcl-2↑	Qin et al. (2016)
					ROS↓, caspase-3↓, Bax↓, p-PKCβII↓, p-p65↓	
Nobiletin	*Citrus depressa* (shiikuwasa)*, Citrus sinensis* (oranges)*,* and *Citrus limon* (lemons)	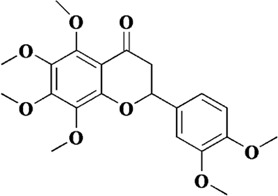	Ox-LDL-induced HUVECs	10–50 μM	ROS↓, MDA↓, TF↓, NF-κB↓	Cirillo et al. (2017)
Oligomeric proanthocyanidins	*Crataegus oxyacantha* berries	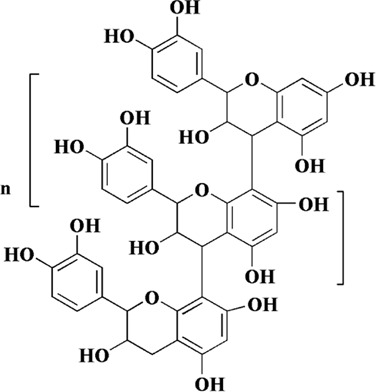	Ox-LDL and C-reactive protein-induced HUVECs	100 μg/mL	NO↑, MMP↑	Jamuna et al. (2022)
					ROS↓, IL-6↓, MCP-1↓, IL-1β↓, LOX-1↓, eNOS↓	
Oolonghomobisflavan A	Leaves of *Camellia sinensis*	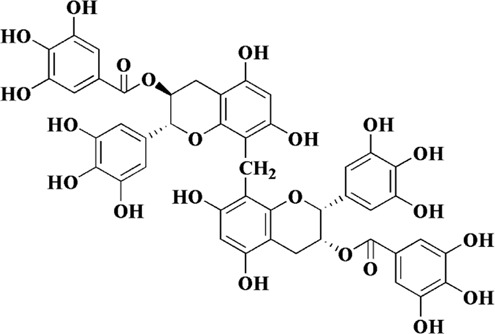	LDL	0.5, 1, 2 μM	Cholesterol ester hydroperoxides↓, thiobarbituric acid reactive substances↓	Sukhbold et al. (2017)
Tricetin	Cereal crops and the pollen of members of the Myrtaceae family	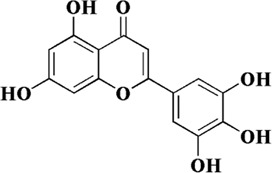	Ox-LDL-induced HUVECs	5, 10 μM	ROS↓, MCP-1↓, IL-1β↓, ICAM-1↓, VCAM-1↓, LOX-1↓, Egr-1↓, ERK1/2↓	Cai et al. (2020)
Vicenin-2	*Cyclopia subternata*	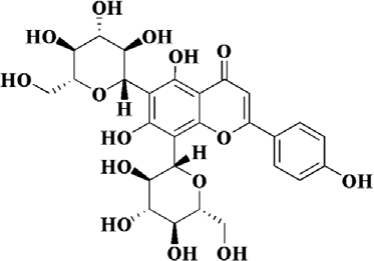	High-glucose-induced HUVECs	20 μM	SOD↑, CAT↑	Ku and Bae (2016)
					ROS↓, MCP-1↓, IL-18↓, ICAM-1↓, VCAM-1↓, NF-κΒ p65↓	
Scolymoside	*Cyclopia subternata*	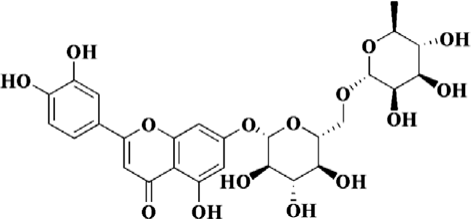	High-glucose-induced HUVECs	20 μM	SOD↑, CAT↑	Ku and Bae (2016)
					ROS↓, MCP-1↓, IL-18↓, ICAM-1↓, VCAM-1↓, NF-κΒ p65↓	

Egr-1, early growth response 1; eNOS, endothelial NO, synthase; ERK1/2, extracellular signal-regulated protein kinase 1 and 2; GPx, glutathione peroxidase; GSH, glutathione; HFD, high-cholesterol diet; HUVECs, Human umbilical vein endothelial cells; ICAM, intercellular adhesion molecule-1; LOX-1, lectin-like ox-LDL, receptor-1; MCP-1, monocyte chemotactic protein-1; MDA, malondialdehyde; MMP, mitochondrial membrane potential; Msr A, methionine sulphoxide reductase; ox-LDL, oxidized low-density lipoprotein; ROS, reactive oxygen species; SD, sprague dawley; SOD, superoxide dismutase; TC, total cholesterol; TF, tissue factor; TG, triglyceride; VCAM-1, vascular cell adhesion molecule-1.

Acacetin, also known as 5, 7-dihydroxy-4′-methoxy flavone, is a monomethoxy flavonoid widely found in medicinal plants such as *Robinia pseudoacacia* L., *Dendranthema morifolium* (Ramat.)Tzvel., and *Saussurea involucrata* (Kar. et Kir.) Sch.-Bip. In nature, acacetin mostly exists in the form of free or glycosides, and has pharmacological activities on cancer, obesity, diabetes, etc (Wu et al., 2022). In recent years, acacetin has been found to have a protective effect on endothelial dysfunction in AS, which has attracted extensive attention from the scientific community. *In vivo* study believed that acacetin significantly accelerated lipid metabolism in AS mice and reduced the levels of inflammatory factors in plasma (Han et al., 2020). *In vitro* experiment confirmed that acacetin could protect mitochondrial function, reverse mitochondrial depolarization, and inhibit the excessive production of ROS and MDA in HUVECs induced by high glucose. On the other hand, the mitoBcl-2/mitoBax ratio in mitochondria was increased after acacetin administration. This protective effect was closely related to the SIRT1-mediated activation of Sirt3/AMPK signaling, and the protein expression of SOD, Bcl-2 and PGC-1α was increased during this process (Han et al., 2020). In addition, the study has shown that acacetin may restore the antioxidant function of endothelial cells by promoting the phosphorylation of Nrf2, the degradation of Keap1 and the expression of methionine sulfite reductase (Wu et al., 2021).

Eupatilin is a flavonoid mainly found in *Artemisia princeps* Pampanini, and also known as 2- (3, 4-dimethoxyphenyl) −5, 7-dihydroxy-6-methoxy-ychromen-4-one. *Artemisia princeps* Pampanini has been widely used as a medicinal plant in Asia over the last thousands of years. In modern times, due to the rapid development of modern pharmacology, eupatilin has been found to have a wider range of pharmacological activities (Lim et al., 2021). For example, eupatilin has therapeutic potential in diseases such as oncology, allergy, and inflammation (Park, 2014; Jeong et al., 2015). In AS, eupatilin has been shown to inhibit the proliferation and migration of human aortic smooth muscle cells. The oxidative stress as well as inflammatory responses occurring in endothelial cells could also be inhibited by eupatilin. In addition, Yu et al. has been confirmed that eupatilin could effectively reduce the ROS content in TNF-α-induced HUVECs, inhibit the expression of VCAM-1 and ICAM-1, and thus reduce the adhesion ability of U937 cells to endothelial cells. The mechanism by which eupatilin exerted its therapeutic effect was closely related to MAPK-NF-ĸB. The phosphorylation of NF-kB p65 and MAPK was significantly inhibited by eupatilin. Taken together, it was suggested that eupatilin could protect endothelial cell function through ROS/MAPK-NF-ĸB (Yu et al., 2015).

From the foregoing, it is known that the preceding flavonoid compounds can protect the cells from oxidative stress damage by restoring the antioxidant capacity of endothelial cells. However, glabridin extracted from the root of *Glycyrrhiza glabra* (licorice) could attenuate the oxidative stress injury to endothelial cells by inhibiting the oxidative sensitivity of LDL. Incubation of LDL with CuSO_4_ or 2,2’ -azobis (2-amidino-propane) dihydrochloride resulted in varying degrees of oxidation of LDL. However, the degree of LDL oxidation was significantly reduced after glabridin treatment, and glabridin inhibited the formation of lipid peroxides and cholesterol linoleic acid hydroperoxides (CLOOH) (Belinky et al., 1998). This protective effect of glabridin provides a novel form of protection for flavonoids. The protective effects of other flavonoid compounds on endothelial cells are shown in Table 1.

## 5 Conclusion and problems

In this review, we summarized the pathogenesis of endothelial dysfunction in AS, and then selected representative flavonoids with anti-oxidative stress effects for relevant elaboration. After summarizing, we have found that flavonoids from natural herbal medicines not only inhibit oxidative stress, but also have anti-inflammatory and anti-adhesion effects in the treatment of endothelial dysfunction. This result is consistent with the multi-level and multi-target advantages of traditional Chinese medicine. In modern clinical practice, it has been demonstrated that flavonoids can be used to reduce the incidence of AS. First of all, epidemiological investigations have shown that increasing the intake of flavonoids in daily diet can effectively reduce the risk of AS (Lagiou et al., 2006; Mursu et al., 2007). Subsequently, more and more evidence has shown that the intake anthocyanins, tea (the main components are flavan-3-ols), *etc.*, can directly reduce the occurrence of AS (Jennings et al., 2012; Ivey et al., 2013). However, after in-depth understanding, flavonoids from natural herbal medicines also have certain limitations and problems that need to be solved urgently. Firstly, most of the models used in the existing studies are *in vitro* models. Flavonoids have been shown to exert protective effects on endothelial cells in experiments, but it is not clear whether this protective effect will change with the transformation of drug structure due to complex changes after drug entry into the body. Secondly, although some researchers have confirmed the protective effect of flavonoids on AS from *in vivo* and *in vitro* experiments, there is no relevant clinical data to support. At the same time, the toxicity and safety of drugs are also essential before the development of drugs. In the case of quercetin, after long-term addition of quercetin to the diet of F344/N rats, there was no obvious toxic damage in the rats at the beginning, but their weight gain was slow and they showed kidney carcinogenic activity in males after 2 years (Dunnick and Hailey, 1992). The oncogenic activity of quercetin remains controversial. However, it is generally believed that quercetin is safe when used under the intended conditions, and caution should be taken when taking quercetin in high doses or for a long time. Therefore, the safety and toxicity of flavonoids should be considered before they are used in clinical practice, and more work needs to be done. Finally, because the flavonoid compounds have more phenolic hydroxyl groups in their structure, it makes their structure unstable. Therefore, it is necessary to consider how to solve the problem of drug stability before developing flavonoid compounds into drugs. Looking at the existing flavonoid drug development, it can be found that the research on the treatment of endothelial dysfunction in AS is still relatively basic, and has not yet considered what kind of preparation the flavonoid is made into, or how it is administered. The development of flavonoids into modern formulations such as nanoparticles may change the instability of the compounds, which can also become the future development direction of flavonoids for the treatment of endothelial dysfunction. In summary, flavonoid compounds hold great promise in the treatment of endothelial dysfunction in AS, but further exploration is needed.
